# Trends of Tobacco and Alcohol Consumption Among People with Diabetes Mellitus in Spain: A Population-Based Study (2014–2020)

**DOI:** 10.3390/epidemiologia7010007

**Published:** 2026-01-04

**Authors:** Luyi Zeng-Zhang, Rodrigo Jiménez-García, Ana López-de-Andrés, Zichen Ji, Jose J. Zamorano-León, Natividad Cuadrado-Corrales, Andrés Bodas-Pinedo, Ana Jiménez-Sierra, Javier de Miguel-Díez

**Affiliations:** 1 Endocrinology and Nutrition Department, Infanta Leonor University Hospital, Universidad Complutense de Madrid, 28031 Madrid, Spain; luyizeng@ucm.es; 2 Department of Public Health and Maternal & Child Health, Faculty of Medicine, Universidad Complutense de Madrid, 28040 Madrid, Spain; josejzam@ucm.es (J.J.Z.-L.); mariancu@ucm.es (N.C.-C.); abodas@ucm.es (A.B.-P.); 3 Department of Public Health and Maternal & Child Health, Faculty of Pharmacy, Universidad Complutense de Madrid, 28040 Madrid, Spain; anailo04@ucm.es; 4 Respiratory Care Department, Hospital Universitario Infanta Leonor, 28031 Madrid, Spain; jizich72@gmail.com; 5 Faculty of Medicine, Universidad San Pablo CEU, 28668 Madrid, Spain; a.jimenez100@usp.ceu.es; 6 Respiratory Care Department, Hospital General Universitario Gregorio Marañón, 28007 Madrid, Spain; javier.miguel@salud.madrid.org

**Keywords:** diabetes mellitus, tobacco consumption, alcohol consumption, Spain, sociodemographic factors, lifestyle, comorbidities

## Abstract

Background/Objectives: To examine trends in tobacco and alcohol consumption among individuals with and without diabetes mellitus (DM) in Spain from 2014 to 2020 and identify sociodemographic, lifestyle, and comorbidity-related predictors of consumption. Methods: Population-based cross-sectional study using data from the 2014 and 2020 European Health Interview Surveys for Spain. Participants’ self-reported tobacco and alcohol consumption were analyzed based on DM status. Results: This study included 7854 participants (3927 participants with DM and 3927 participants without DM). Among participants with DM, tobacco and alcohol consumption remained stable over the study period, with tobacco from 15.2% in 2014 to 14.8% in 2020 (*p* = 0.761) and alcohol from 37.2% to 39.8% (*p* = 0.088), respectively. Tobacco consumption did not differ significantly between those with and without DM (15.0% vs. 15.2%, *p* = 0.777). However, alcohol consumption was significantly lower among those with than without DM (38.6% vs. 48.7%, *p* < 0.001). In those with DM, predictors of tobacco consumption included male sex, younger age, alcohol consumption, living without a partner, and DM, and predictors of alcohol consumption included male sex, active smoking, higher education, and sedentary lifestyle. Conclusions: Between 2014 and 2020, both tobacco and alcohol consumption remained stable among individuals with DM. The prevalence of alcohol consumption was lower among those with than without DM. Key predictors of tobacco and alcohol consumption included sex, lifestyle behaviors, and socioeconomic factors. These findings highlight the need for targeted public health interventions to reduce harmful substance use in DM populations and mitigate associated health risks.

## 1. Introduction

Diabetes mellitus (DM) is a highly prevalent chronic disease and one of the leading causes of morbidity and mortality globally. Representing a serious public health issue, the prevalence of DM in Spain reached 14.8% in 2021, a 42% increase since 2019 [1], and a concomitant increase in associated complications has contributed to higher premature mortality rates [2].

In this context, unhealthy behaviors play a central role in the development of chronic diseases, including hypertension, type 2 DM, and coronary artery disease [3]. These conditions are often largely driven by modifiable lifestyle factors, including poor diet, physical inactivity, tobacco and alcohol use, insufficient sleep, and chronic stress, which despite advances in pharmacological therapies, remain inadequately addressed in mainstream healthcare systems [4].

Among these modifiable behaviors, tobacco and alcohol consumption have been consistently linked to both the incidence and progression of DM [5,6]. These habits not only directly affect the pathophysiology of DM but also contribute to the development of other complications and comorbidities, such as hypertension, dyslipidemia, and obesity, significantly increasing cardiovascular risk and overall mortality [7,8].

As potent aggravators of insulin resistance, nicotine and other compounds present in cigarette smoke induce a state of chronic oxidative stress that damages the pancreatic beta cells responsible for insulin production [8,9]. The Insulin Resistance Atherosclerosis Study, a 5-year prospective study, analyzed the relationship between tobacco consumption and the incidence of DM. It observed a DM incidence of 25% among active smokers compared with 14% among non-smokers, and the probability of developing DM in smokers was 2.66-fold higher than in non-smokers [10]. In another prospective study, among smokers who consumed more than two packs per day, men had a 45% higher risk of developing DM compared with non-smokers, while this risk in women was 74% [11]. In addition, the NutriNet-Santé study conducted in France observed a 36% higher risk of incidence of type 2 DM among former or active smokers compared with those who had never smoked [12]. The risk doubled in those who smoked heavily compared with those who smoked slightly [12].

In addition to contributing to the onset of DM, smoking is also associated with higher risks of cardiovascular events, premature death, microvascular complications, and poor glycemic control [13,14]. Individuals who smoked 15–30 pack-years or more than 30 pack-years had a probability of proteinuria of 2.78 and 3.2, respectively [15]. A higher risk of diabetic retinopathy and proliferative retinopathy was observed among smokers than non-smokers [16]. Several studies have also demonstrated an increased risk of cardiovascular disease and coronary heart disease among smokers with DM [6].

In contrast to smoking, the effects of alcohol consumption on DM vary according to the quantity and pattern of consumption [17]. Several studies have shown the protective effect of moderate alcohol consumption against the development of DM [18,19]. In a prospective study with a 10-year follow-up, in which individuals were categorized according to alcohol consumption as abstinent or low, moderate, or high consumers, the 10-year incidence of DM was 13.4% in men and 12.4% in women. After adjustment, it was found that those who consumed up to one alcoholic drink per day had a 53% lower risk of developing DM compared with abstainers [18]. Furthermore, trend analysis showed a significant U-shaped relationship between the amount of alcohol consumed and the incidence of DM, indicating that both abstinence and high consumption are associated with a higher risk, whereas low consumption is associated with the lowest risk [20]. A meta-analysis reported similar findings, identifying a U-shaped relationship for both sexes [19].

However, excessive alcohol consumption has adverse metabolic effects, disrupting hepatic glucose metabolism and promoting both hypoglycemia and hyperglycemia, thus complicating glycemic control in individuals with DM [21,22]. Excessive alcohol intake also increases the risk of pancreatitis, which can irreversibly damage the pancreatic beta cells, thereby increasing DM incidence or accelerating its progression [23].

From a population perspective, trends in tobacco and alcohol consumption provide important contextual information. In the general Spanish population, the period between 2014 and 2020 has been described as relatively stable in terms of overall tobacco and alcohol consumption [24,25]. Within this context, it is relevant to examine whether similar stability was observed among individuals with DM during this period. With respect to tobacco control, law 42/2010 constituted the main regulatory framework in Spain throughout these years. Although tobacco taxation increased in 2016, tax levels remained low in comparison with other European countries. Moreover, restrictions on smoking in outdoor public spaces had already been implemented before 2014 [24].

In the case of alcohol consumption, a 5% increase in alcohol taxes was implemented in 2016, and throughout the 2010s the National Plan on Drugs strengthened campaigns promoting moderate consumption, preventive education among young people, policies addressing alcohol and driving, and the detection and treatment of alcohol consumption in primary care [25]. The latter part of the study period overlaps with the onset of the COVID-19 pandemic, which has been shown to affect alcohol and tobacco consumption patterns differently across countries [26,27]. This exceptional context further underscores the relevance of assessing temporal changes in consumption behaviors within the population with DM.

For individualized management, it is essential to recognize that the relationship between substance consumption and DM is influenced by multiple sociodemographic factors, such as age, sex, and education level, as well as lifestyle factors, including diet and physical activity levels [5,21]. In clinical practice, understanding this relationship is crucial for optimizing the management of the disease and unhealthy habits. Identifying the risk factors associated with substance use among individuals with DM will also facilitate the design of intervention strategies aimed at promoting cessation.

This study aimed to examine the relationship between tobacco and alcohol consumption and DM status between 2014 and 2020 in Spain. Data from the 2014 and 2020 European Health Interview Surveys for Spain (EHISS) was correlated with sociodemographic factors, lifestyle behaviors, and associated comorbidities to identify modifiable risk factors for substance use in individuals with DM.

## 2. Materials and Methods

### 2.1. Study Design

This population-based cross-sectional study used data from the 2014 and 2020 EHISS. Conducted by the Spanish National Statistics Institute (INE) in collaboration with the Ministry of Health, the EHISS aims to collect information on the health status of the population residing in Spain, including health determinants and the use of healthcare resources [28].

The EHISS is conducted periodically and targets those aged ≥15 years. Data are collected through in-person interviews at participants’ homes, except for some telephone interviews between March and July 2020 due to the COVID-19 lockdown. The 2014 and 2020 EHISS surveys used a standardized questionnaire developed and validated by Eurostat within the framework of Regulation (EU) 2018/255. Its content is based on a prior methodological review assessing the quality and reliability of the instruments, including the sections on tobacco and alcohol. Data collection was subject to quality controls established by the National Statistics Institute (INE), with periodic field inspections. Methodological consistency across different editions ensures comparability of results [28].

### 2.2. Participants with and Without Diabetes Mellitus

Survey participants were selected through probabilistic sampling. In the first stage, geographic areas were selected by stratified random sampling, followed by randomly selecting households and then individuals within those households, ensuring representative results for the resident Spanish population [28].

Participants with DM were defined as individuals who reported having been diagnosed with DM by a physician. Each participant with DM was matched with a control without a DM diagnosis, matched by sex, age, EHISS edition, and place of residence. If more than one control was identified per case, the selection was made randomly.

### 2.3. Variables

The dependent variables were tobacco and alcohol consumption. Participants who answered affirmatively to the question “Could you tell me if you smoke?” were classified as active smokers. Alcohol consumption was considered positive for participants who reported drinking alcohol at least once a month. Alcohol and tobacco variables were dichotomized for analysis. Alternative categorizations were considered, but missing data for alcohol quantity and small sample sizes for tobacco subgroups prevented reliable multi-category analyses.

The independent variables were classified into three groups:

- Sociodemographic variables: sex, age, education level, living with a partner, and survey edition.
- Lifestyle variables: body mass index (BMI), self-perceived health, sedentary behavior, and history of accidents.
- Comorbidity variables: chronic obstructive pulmonary disease (COPD), asthma, myocardial infarction, stroke, cancer, mental illness, hypertension, dyslipidemia, chronic pain, osteoporosis, peptic ulcer, and periodontal disease.

The questions used to create the study variables and their categorization based on possible responses are described in detail in Table S1.

### 2.4. Statistical Analysis

Sociodemographic, lifestyle, and comorbidity variables were descriptively analyzed. Normally distributed quantitative variables are presented as the mean ± standard deviation (SD), and non-normally distributed variables are presented as the median (interquartile range [IQR]). Qualitative variables are reported as the frequency (percentage).

Quantitative variables were compared between groups using Student’s t-test or the Mann–Whitney U test, depending on the normality of their distribution, and qualitative variables were compared using the chi-square test. The association between tobacco and alcohol consumption and DM was evaluated using two logistic regression models, adjusted for variables with significant associations in bivariate analyses or deemed relevant in previous studies. The results are presented as odds ratios (ORs) with 95% confidence intervals (CIs).

A two-tailed *p*-value of <0.05 was considered statistically significant. All analyses were performed using SPSS (version 26.0; IBM Corp., Armonk, NY, USA).

### 2.5. Ethical Considerations

This study was based on data obtained from the EHISS, which ensures participant confidentiality and anonymity according to current ethical and legal regulations. Since the data were anonymized and did not contain information that could identify individuals, approval by a Research Ethics Committee or informed consent from participants was not required. The EHISS datasets can be freely downloaded from the INE website [28].

## 3. Results

This study included 7854 participants, comprising 3927 with DM and 3927 without DM. Among them, 3824 (48.7%) were male, the mean age was 68.8 ± 13.1 years, and the median BMI was 26.9 kg/m^2^ (24.4–30.2). Regarding tobacco and alcohol consumption, 1185 participants (15.1%) reported being active smokers, and 3428 (43.6%) reported consuming alcohol.

**Figure 1:**
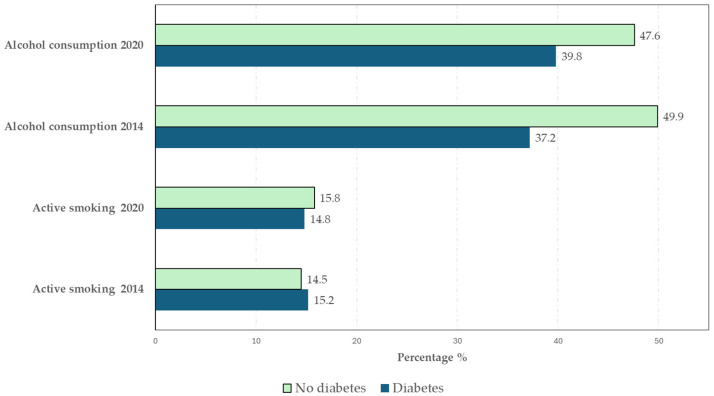
Prevalence of active smoking and alcohol consumption in participants with and without diabetes according to the European Health Interview Surveys for Spain conducted in 2014 and 2020.

Figure 1 presents the prevalence of tobacco and alcohol consumption among participants with and without DM by survey year. Tobacco and alcohol consumption remained largely stable among participants with DM between 2014 and 2020, with no significant changes observed. Tobacco use decreased slightly from 15.2% to 14.8% (*p* = 0.761), while alcohol consumption increased modestly from 37.2% to 39.8% (*p* = 0.088). Similarly, no significant changes in tobacco or alcohol consumption were observed among participants without DM over the same period. Detailed 95% confidence intervals for all variables can be found in Supplementary Table S3.

Table S2 presents the distribution of participants with DM according to the 2014 and 2020 EHISS: 1874 (47.7%) were from the 2014 EHISS, and 2053 (52.2%) were from the 2020 EHISS. The percentage of individuals with primary education was higher in the 2014 EHISS (32.2%) than in the 2020 EHISS (24.5%; *p* < 0.001). Conversely, the percentage of participants with higher education was higher in the EHISS 2020 than in the 2014 EHISS. Regarding self-rated health, the percentage of participants reporting an unfavorable perception of their health status was higher in the 2014 EHISS (66.25%) than in the 2020 EHISS (60.0%; *p* < 0.001). Additionally, the prevalence of obesity among participants with DM was higher in the 2014 EHISS (35.0%) than in the 2020 EHISS (30.0%; *p* = 0.009). Regarding comorbidities, the percentages of participants with COPD, asthma, mental illness, chronic pain, peptic ulcer, periodontal disease, and accident history were higher in the EHISS 2014 than in the 2020 EHISS.

### 3.1. Prevalence of Tobacco and Alcohol Consumption Among Participants with and Without DM, Overall and by Sociodemographic Variables

Table 1 shows the prevalence of tobacco and alcohol consumption among participants with and without DM in the 2014 and 2020 EHISS, overall and by sociodemographic variables.

Overall, tobacco consumption did not differ significantly between participants with and without DM (15.0% vs. 15.2%, *p* = 0.777). However, among participants not living with a partner, tobacco consumption was significantly more common in the group without DM (15.8%) compared to the group with DM (13.4%; *p* = 0.042). In addition, tobacco consumption was more common among men, those aged 50–69 years, and those with secondary or higher education.

Among participants without DM, tobacco consumption was significantly associated with variables such as sex, age group, and education level. Among participants with DM, in addition to these variables, tobacco consumption was also significantly associated with the variable of living with a partner.

Alcohol consumption was significantly more common among participants without DM compared to those with DM (48.7% vs. 38.6%, *p* < 0.001) and across most analyzed variables (gender, age group, education level, and living with a partner), except for age > 80 years, where the difference was not statistically significant. Alcohol consumption was more common among males, those with secondary or higher education, and those living with a partner.

Among participants with and without DM, alcohol consumption was significantly associated with sex, age group, education level, and living with a partner.

### 3.2. Prevalence of Tobacco and Alcohol Consumption Among Participants with and Without DM by Lifestyle Variables

Table 2 presents the prevalence of tobacco and alcohol consumption among participants with and without DM according to lifestyle-related variables.

Tobacco consumption did not differ significantly between participants with and without DM for most lifestyle variables. However, tobacco consumption was significantly higher among individuals with DM (16.5%) compared to those without DM (12.1%) within the subgroup of participants with a BMI in the obese range (*p* = 0.008). Additionally, alcohol consumption, self-rated health, and BMI were significantly associated with tobacco consumption in both groups, with and without DM.

Significant differences were identified in most of these variables, notably higher alcohol consumption among individuals without DM, except in the subgroup of participants with an unfavorable self-rated of health status and those with a BMI in the underweight range. Alcohol consumption was significantly associated with tobacco consumption, self-rated health, sedentary lifestyle, and a history of accidents in both groups. Among participants without DM, alcohol consumption was also significantly associated with BMI.

### 3.3. Prevalence of Tobacco and Alcohol Consumption in Participants with and Without DM by Comorbidity Variables

Table 3 presents the prevalence of tobacco and alcohol consumption based on comorbidities in participants with and without DM. Tobacco consumption differed significantly between participants with and without DM only for mental illness (16.9% vs. 12.5%, *p* = 0.019) and hypertension (12.1% vs. 9.6%, *p* = 0.011).

Among participants without DM, tobacco consumption was significantly associated with a history of myocardial infarction, cancer, mental illness, hypertension, dyslipidemia, chronic pain, and osteoporosis. These associations were also observed among participants with DM, except for mental illness, and a significant association was also found for periodontal disease.

Among participants without DM, alcohol consumption was more common among those who reported nearly all comorbidities except for stroke, cancer, and peptic ulcer disease.

Among participants without DM, alcohol consumption was significantly associated with comorbidities such as stroke, cancer, mental illness, hypertension, chronic pain, and osteoporosis. Among participants with DM, alcohol consumption was significantly associated with COPD, asthma, myocardial infarction, stroke, mental illness, hypertension, chronic pain, and osteoporosis.

### 3.4. Predictors of Tobacco Consumption by DM Status

Table 4 presents factors associated with tobacco consumption by DM status in multivariable analyses. Female sex, age > 60 years, sedentary lifestyle, and having asthma or hypertension were significantly associated with a lower prevalence of tobacco consumption in both participants with and without DM. Alcohol consumption, not living with a partner, and having a history of COPD were significantly associated with higher tobacco consumption in both groups.

In the joint analysis of both groups, secondary education (OR: 1.28, 95% CI: 1.02–1.60), a history of DM (OR: 1.15, 95% CI: 1.01–1.34), and mental illness (OR: 1.29, 95% CI: 1.06–1.57) were identified as predictors of higher tobacco consumption. The latter was also identified as a significant predictor of higher tobacco consumption among participants with DM.

### 3.5. Predictors of Alcohol Consumption by DM Status

Table 5 presents the factors associated with alcohol consumption by DM status. Female sex and having a more unfavorable self-rated of health status were associated with a lower prevalence of alcohol consumption in both participants with and without DM. Among participants without DM, age > 80 years (OR: 0.64, 95% CI: 0.46–0.90) and having participated in the 2020 EHISS (OR: 0.82, 95% CI: 0.70–0.95) were negatively associated with alcohol consumption. Among participants with DM, a history of myocardial infarction (OR: 0.71, 95% CI: 0.55–0.90) and stroke (OR: 0.59, 95% CI: 0.42–0.83) were negatively associated with alcohol consumption. In the joint analysis, a history of DM was negatively associated with lower alcohol consumption (OR: 0.70, 95% CI: 0.63–0.78).

Conversely, tobacco consumption, sedentary lifestyle, and secondary or higher education were associated with higher alcohol consumption in both groups. Additionally, among participants without DM, a history of asthma (OR: 1.45, 95% CI: 1.04–2.02) was positively associated with alcohol consumption. Among participant with DM, being aged 60–69 years (OR: 1.47, 95% CI: 1.09–1.97) was positively associated with higher alcohol consumption.

## 4. Discussion

### 4.1. Temporal Evolution of Tobacco and Alcohol Consumption

Several epidemiological studies have examined the prevalence of tobacco use among people with DM. In our study, the prevalence of tobacco consumption among individuals with DM was 15.2% in 2014 and 14.8% in 2020, which aligns with Rodrick et al. who reported lower smoking rates among individuals with DM in Spain (15.6%) compared to the global estimate (20.8%) [29]. However, another cross-sectional study carried out in 27 European countries between 2019 and 2020 showed an even lower current smoking prevalence of 12.2% overall and 8.9% in Spain among individuals aged over 50 years with DM [30].

In our study, we observed no significant changes in tobacco use between 2014 and 2020. In contrast, a study by Dorner et al. between 2007 and 2014 in Austria observed an increase in smoking among women with DM, while the prevalence among men remained stable [31]. This may indicate that public health efforts to reduce substance use in individuals with DM are not fully effective, or that patients and healthcare providers may not prioritize these behaviors. In the same way, tobacco control policies in Spain present several limitations compared with countries that have made more progress in this area. Taxation is insufficient, prices are among the lowest in Europe, and the tax burden is lower for products such as roll-your-own tobacco or electronic cigarettes. In addition, the regulation of outdoor spaces is limited and less ambitious than in places such as California [24].

Regarding alcohol consumption, a study in the United States reported a prevalence of 63.6% among men and 44.9% among women with DM [32]. In our study, the prevalence of alcohol consumption in individuals with DM was lower and remained stable from 2014 to 2020. This is consistent with another study evaluating long-term trends in alcohol use among the Spanish population, which observed a 4.5% annual decrease from 2006 to 2011, followed by a period of stability from 2011 onwards [25]. This change may be explained by a shift in drinking patterns, whereby beer has replaced wine as the preferred alcoholic beverage, and binge drinking among young people is increasingly replacing the daily alcohol consumption among adults. The change may also be associated with the implementation of public health measures by the Spanish government, such as raising the minimum legal age for purchasing alcohol, introducing advertising regulations, and prohibiting the sale of alcohol in public educational institutions and at sporting events [25].

It is relevant that Spain entered lockdown on March 15 of 2020, which coincided with the final quarter of the data collection period of the 2020 survey. This represents an important change in conditions that could have modified lifestyle behaviors and influenced the observed prevalence of tobacco and alcohol consumption during this period. Two studies conducted in Spain evaluating unhealthy lifestyle behaviors during lockdown, including tobacco and alcohol use, observed a trend toward reduced consumption during the confinement period [27,33]. These findings suggest that the population tended to take greater care of their health by avoiding unhealthy lifestyle behaviors during the pandemic. However, studies from other countries reported different results. For example, in Brazil, rates of alcohol consumption and smoking increased by approximately 20% and 30%, respectively, during the COVID-19 pandemic [26]. This highlights the need for further studies conducted in Spain to better assess the effect of the pandemic on tobacco and alcohol consumption during this period.

### 4.2. Tobacco and Alcohol Consumption by Sociodemographic, Lifestyle, and Comorbidity Variables

Our study found no significant difference in the prevalence of tobacco consumption between individuals with and without DM. From a clinical perspective, a lower prevalence of tobacco consumption would be expected among individuals with DM due to the elevated risk of vascular complications associated with smoking. This finding contradicts a previous study conducted in the United States from 2001 to 2020 that examined trends in the prevalence of smoking among individuals with and without DM. A 9% lower prevalence of smoking and a 13% higher prevalence of attempts to quit smoking were reported among adults with DM compared to those without, possibly due to a greater awareness of smoking risks in this population [34].

Similarly, tobacco consumption was higher among individuals with DM living with obesity or hypertension, highlighting the interaction between various cardiovascular risk factors [35]. The coexistence of these factors not only increases the risk of metabolic and cardiovascular complications but may also amplify their adverse effects through synergistic interactions [6,7]. Moreover, the relationship observed between mental illness and tobacco consumption among individuals with DM in our study aligns with prior research [36]. This link could be explained by the greater psychological vulnerability of individuals with DM, who often face chronic stress related to disease management. This stress can trigger or exacerbate mental disorders, thereby increasing the likelihood of using tobacco as an emotional coping mechanism.

In terms of alcohol consumption, our study found that individuals with DM had lower prevalence than those without DM, both overall and across most examined categories. This result is consistent with previous studies documenting an inverse relationship between DM and alcohol consumption [21,37]. The lower alcohol consumption observed among individuals with DM compared with the non-diabetic population may reflect a combination of factors. A diagnosis of DM and ongoing contact with healthcare services may lead to behavioral changes aimed at reducing alcohol intake, particularly due to concerns about hypoglycemia, especially among individuals treated with insulin or sulfonylureas. In addition, reducing or stopping alcohol consumption may be easier to achieve than smoking cessation, which could partly explain the greater reduction in alcohol use. Finally, the role of information bias should be considered, as individuals with DM may report lower alcohol consumption due to fear of criticism or social judgment [21,37].

### 4.3. Predictors of Tobacco and Alcohol Consumption in Individuals with DM

Our study identified a history of DM as a predictor of higher tobacco consumption, which is significant given the adverse effects of smoking on cardiovascular risk. In this context, previous studies have highlighted the diabetogenic effect of smoking, identifying it as a risk factor for developing type 2 DM [13,38,39,40]. This finding reinforces the need to prioritize intervention strategies aimed at smoking cessation in the general population, particularly among individuals with DM. In addition, among individuals with DM, factors such as male sex, younger age, and alcohol consumption were positively associated with higher tobacco consumption. This finding may be related to cultural patterns and social pressures [41,42].

However, the association between sedentary lifestyle and lower tobacco consumption was unexpected, contradicting prior studies linking sedentary lifestyles with unhealthy behaviors, including smoking [43,44]. Therefore, this finding requires further study to interpret it correctly. Regarding social factors, not living with a partner was positively associated with tobacco consumption, consistent with research highlighting the emotional and social support provided by a partner as protective against smoking [45]. Nevertheless, it has also been reported that living with a smoking partner could increase the likelihood of maintaining this habit [46,47].

Among the examined comorbidities, an inverse association was identified between hypertension and tobacco consumption, which is unexpected given that smoking is a widely recognized risk factor for hypertension [48]. This finding may be explained by the motivation that a hypertension diagnosis gives patients to reduce or quit smoking due to medical interventions or increased perception of associated risks [49]. Similarly, mental illness was also associated with tobacco use among individuals with DM, consistent with the bidirectional relationship previously described between these three conditions [50,51]. In a cross-sectional study examining the determinants of anxiety and depression symptoms in patients with DM, diabetic complications, smoking, uncertainty regarding glycemic control, and being an ex-drinker or heavy drinker were identified as risk factors for higher anxiety and depression scores [50]. Furthermore, in another study by Clyde et al. that investigated the association between depression and smoking status in patients with type 2 DM, a significant relationship was found between moderate-to-heavy smoking and depression [51]. Moreover, DM is frequently associated with higher stress levels, as individuals must continuously monitor their diet, physical activity, treatment, and glycemic control. These ongoing demands can increase disease-related distress, make smoking cessation more challenging and elevating the risk of anxiety and depression [52].

With respect to respiratory conditions, COPD was positively associated with tobacco consumption, consistent with its pathophysiology related to chronic smoke exposure [53,54]. Conversely, asthma was inversely associated with tobacco consumption, likely because individuals with asthma tend to avoid tobacco smoke to reduce the risk of acute exacerbation [55].

Regarding alcohol consumption, a history of DM was identified as a predictor of lower alcohol consumption, potentially reflecting greater adherence to medical recommendations and growing awareness about metabolic complications in this population [21,37]. However, the relationship between alcohol consumption and the risk of developing DM remains controversial. While some studies have indicated that moderate alcohol consumption could reduce the risk of type 2 DM [56,57,58], others have found insufficient evidence to support this relationship [59,60]. These discrepancies could be due to differences in methodological designs and study populations, suggesting the need for further research.

Factors such as male sex and being an active smoker were associated with higher alcohol consumption, consistent with data from previous studies [61,62]. An association was also observed between higher educational attainment and greater alcohol consumption, which could be explained by the previously documented link between higher socioeconomic status and greater access to alcohol [63,64,65]. However, other studies suggest an inverse relationship, linking alcohol consumption to lower educational levels [66,67], indicating that the relationship may vary depending on the study population.

Furthermore, unfavorable self-rated health status and sedentary lifestyle were associated with higher alcohol consumption. These findings are consistent with prior studies in which these factors were linked to less healthy lifestyles and may reflect a lack of effective mechanisms for stress management or improving quality of life [62,68,69]. Finally, among the examined comorbidities, a history of myocardial infarction or stroke was inversely associated with alcohol consumption, highlighting the impact of comorbidities on the adoption of healthier lifestyles [70,71].

### 4.4. Practical Implications

According to the latest American Diabetes Association guidelines, people with DM should routinely receive counseling about tobacco and vaping product use. They should be encouraged to quit smoking and vaping, and clinicians should offer advice or pharmacological treatments that can help with cessation. Guidelines also recommend limiting or avoiding alcohol consumption, noting the risk of hypoglycemia, hyperglycemia, and weight gain associated with alcohol use [7].

For physicians, it is important to identify the use of these substances among people with DM, especially in those with other unhealthy lifestyle behaviors, such as physical inactivity, or related comorbidities. Education and support in abandoning these harmful habits should be offered.

### 4.5. Strengths and Limitations

Our study had several strengths. First, its sample size was large, resulting in high statistical power that allowed the detection of significance in small differences. Second, the EHISS methodology is rigorous and validated, and its uniformity permits comparisons across different editions. Third, the anonymous format of the EHISS enables the collection of more reliable data. Finally, the EHISS collects sociodemographic and lifestyle data that are generally not recorded in medical histories.

However, our study also had several limitations. First, the lack of classification by type of DM represents an important limitation of our study, as individuals with type 1 and type 2 DM differ in disease course and clinical behavior due to their distinct pathophysiological mechanisms. The risk of complications related to tobacco and alcohol consumption also differs between these groups. In addition, information on disease duration was not available, a factor that may influence the degree of adherence to healthy lifestyle recommendations. The lack of information on treatment, complications, and level of metabolic control limits the evaluation of the association between tobacco and alcohol consumption and these factors, thus reducing the clinical applicability of the results. This points to the need for further prospective studies to adequately assess these aspects. Second, the DM diagnosis recorded in the EHISS was self-declared by participants, which could introduce self-report bias and potential misclassification in diagnostic validity, and which could lead to under- or overestimation of the associations observed. Third, data related to the quantity and patterns of tobacco and alcohol consumption were not collected. This represents a limitation for interpreting the study findings, as the risks of developing DM and its complications are typically dependent on the level of consumption. Fourth, as participants answered the EHISS retrospectively, memory bias could exist. Fifth, the response rates in the EHISS (74.6% in 2014 and 72.2% in 2020) [72,73] and potential bias arising from non-participation must be considered. Sampling weights were not applied in the data analysis, which may affect the generalizability of our results to the broader Spanish context. Finally, due to the pandemic, some participants in the 2020 survey were interviewed by telephone. As telephone responses may not have the same reliability as those obtained in face-to-face interviews, the comparability of the data may be reduced.

## 5. Conclusions

Between 2014 and 2020, both tobacco and alcohol consumption remained relatively stable among individuals with DM. While no significant difference in tobacco consumption was found between individuals with and without DM, lower alcohol consumption was evident among those with DM. The study also identifies predictors of tobacco consumption (male sex, younger age, alcohol consumption, not living with a partner, and history of DM) and alcohol consumption (male sex, active smoker, higher educational attainment, and sedentary lifestyle). These findings enable the design of specific intervention strategies to promote the adoption of healthy habits in the DM population.

## Figures and Tables

**Table 1:** Prevalence of active smoking and alcohol consumption among participants with and without diabetes globally and according to sociodemographic variables in the European Health Interview Surveys for Spain conducted in 2014 and 2020.

		Active Smoking					Alcohol Consumption				
Variable	Categories	No Diabetes		Diabetes		*p* Value	No Diabetes		Diabetes		*p* Value
		n	%	n	%		n	%	n	%	
Total	Total	597	15.2	588	15.0	0.777	1913	48.7	1515	38.6	<0.001
Sex ^a,b,c,d^	Male	399	20.9	386	20.2	0.603	1281	67.0	1110	58.1	<0.001
	Female	198	9.8	202	10.0	0.833	632	31.4	405	20.1	<0.001
Age groups ^a,b,c,d^	≤9	90	28.4	104	32.8	0.228	185	58.4	133	42.0	<0.001
	50–59	181	32.7	169	30.5	0.438	339	61.2	249	44.9	<0.001
	60–69	199	20.1	193	19.5	0.735	589	59.6	483	48.9	<0.001
	70–79	101	8.6	97	8.3	0.766	553	47.3	438	37.5	<0.001
	≥0	26	2.9	25	2.8	0.877	247	27.5	212	23.6	0.058
Educational level ^a,b,c,d^	No studies/primary	56	6.7	78	7.0	0.754	251	30.0	268	24.2	0.005
	Secondary	446	17.4	443	17.8	0.711	1317	51.4	1065	42.8	<0.001
	High education	95	18.0	67	20.1	0.437	345	65.3	182	54.7	0.002
Living with a partner ^b,c,d^	No	281	15.8	241	13.4	0.042	743	41.7	564	31.3	<0.001
	Yes	316	14.7	347	16.3	0.150	1170	54.5	951	44.8	<0.001

*p* value for difference between participants with diabetes and diabetes age and sex matched controls. ^a^ significant association between the variable and Active Smoking among participants without Diabetes. ^b^ Significant association between the variable and Active Smoking among participants with Diabetes. ^c^ Significant association between the variable and Alcohol consumption among participants without Diabetes. ^d^ Significant association between the variable and Alcohol consumption among participants with Diabetes.

**Table 2:** Prevalence of active smoking and alcohol consumption among participants with and without diabetes in the European Health Interview Surveys for Spain (EHISS) conducted in 2014 and 2020, according to lifestyle variables.

		Active Smoking					Alcohol Consumption				
Variable	Categories	No Diabetes		Diabetes		*p* Value	No Diabetes		Diabetes		*p* Value
		n	%	n	%		n	%	n	%	
Alcohol consumption ^a,b^	No	200	9.9	265	11.0	0.254	-	-	-	-	-
	Yes	397	20.8	323	21.3	0.685	-	-	-	-	-
Active Smoking ^c,d^	No	-	-	-	-	-	1516	45.5	1192	35.7	<0.001
	Yes	-	-	-	-	-	397	66.5	323	54.9	<0.001
Self-rated health ^a,b,c,d^	Fair/poor/very poor	203	11.8	328	13.3	0.172	578	33.7	773	31.3	0.097
	Very good/good	394	17.8	260	17.9	0.967	1335	60.3	742	51.0	<0.001
Body mass index category ^a,b,c^	Underweight < 18.5	9	19.1	2	11.1	0.439	14	29.8	5	27.8	0.873
	Normal weight 18.5–24.9	268	20.8	159	18.2	0.145	650	50.4	324	37.2	<0.001
	Overweight 25.0–29.9	206	12.7	215	13.7	0.414	845	52.3	657	42.0	<0.001
	Obesity ≥30	85	12.1	194	16.5	0.008	323	45.8	458	39.0	0.004
Sedentary lifestyle ^c,d^	No	354	15.3	307	15.7	0.758	1330	57.7	924	47.2	<0.001
	Yes	243	15.0	281	14.3	0.534	583	36.0	591	30.0	<0.001
Accident ^c,d^	No	558	15.5	537	15.2	0.676	1787	49.7	1407	39.7	<0.001
	Yes	39	11.7	51	13.2	0.553	126	37.8	108	27.9	0.005
EHISS	2014	272	14.5	284	15.2	0.581	936	49.9	697	37.2	<0.001
	2020	325	15.8	304	14.8	0.363	977	47.6	818	39.8	<0.001

*p* value for difference between participants with diabetes and diabetes age and sex matched controls. ^a^ Significant association between the variable and Active Smoking among participants without Diabetes. ^b^ Significant association between the variable and Active Smoking among participants with Diabetes. ^c^ Significant association between the variable and Alcohol consumption among participants without Diabetes. ^d^ Significant association between the variable and Alcohol consumption among participants with Diabetes.

**Table 3:** Prevalence of active smoking and alcohol consumption among participants with and without diabetes in the European Health Interview Surveys for Spain conducted in 2014 and 2020, according to clinical variables.

		Active Smoking					Alcohol Consumption				
Variable	Categories	No Diabetes		Diabetes		*p* Value	No Diabetes		Diabetes		*p* Value
		n	%	n	%		n	%	n	%	
COPD ^d^	No	556	15.2	521	14.8	0.679	1801	49.1	1376	39.1	<0.001
	Yes	41	15.8	67	16.3	0.844	112	43.1	139	33.9	0.017
Asthma ^d^	No	573	15.4	554	15.2	0.808	1817	48.9	1426	39.2	<0.001
	Yes	24	11.4	34	11.9	0.860	96	45.5	89	31.1	0.001
Cardiac ischemia ^a,b,d^	No	575	15.7	533	15.4	0.758	1796	49.0	1360	39.4	<0.001
	Yes	22	8.4	55	11.5	0.182	117	44.7	155	32.5	0.001
Stroke ^c,d^	No	582	15.4	559	15.2	0.842	1873	49.4	1456	39.6	<0.001
	Yes	15	10.9	29	11.7	0.815	40	29.2	59	23.9	0.254
Cancer ^a,b,c^	No	568	15.6	558	15.5	0.878	1797	49.3	1400	38.8	<0.001
	Yes	29	10.2	30	9.4	0.749	116	40.8	115	36.2	0.238
Mental disease ^a,c,d^	No	520	15.7	447	14.4	0.157	1706	51.5	1287	41.6	<0.001
	Yes	77	12.5	141	16.9	0.019	207	33.5	228	27.4	0.012
High blood pressure ^a,b,c,d^	No	438	19.3	290	19.7	0.746	1185	52.2	616	41.9	<0.001
	Yes	159	9.6	298	12.1	0.011	728	43.9	899	36.6	<0.001
Dyslipidemia ^a,b^	No	444	16.4	320	16.8	0.748	1344	49.7	750	39.3	<0.001
	Yes	153	12.5	268	13.3	0.536	569	46.6	765	37.9	<0.001
Pain ^a,b,c,d^	No	419	16.4	370	16.1	0.757	1364	53.3	1001	43.4	<0.001
	Yes	178	13.0	218	13.4	0.725	549	40.1	514	31.7	<0.001
Osteoporosis ^a,b,c,d^	No	569	16.0	561	15.8	0.823	1803	50.7	1431	40.3	<0.001
	Yes	28	7.5	27	7.1	0.832	110	29.5	84	22.1	0.021
Gastric or duodenal ulcer	No	572	15.4	548	14.9	0.558	1812	48.9	1415	38.6	<0.001
	Yes	25	11.3	40	15.3	0.192	101	45.5	100	38.3	0.111
Periodontal disease ^b^	No	220	14.5	175	12.9	0.199	714	47.2	517	38.0	<0.001
	Yes	377	15.6	413	16.1	0.658	1199	49.7	998	38.9	<0.001

*p* value for difference between participants with diabetes and diabetes age and sex matched controls. ^a^ Significant association between the variable and Active Smoking among participants without Diabetes. ^b^ Significant association between the variable and Active Smoking among participants with Diabetes. ^c^ Significant association between the variable and Alcohol consumption among participants without Diabetes. ^d^ Significant association between the variable and Alcohol consumption among participants with Diabetes. COPD: Chronic obstructive pulmonary disease.

**Table 4:** Predictors of active smoking in the population participating in the European Health Interview Surveys (2014 and 2020) in Spain, according to the presence of diabetes.

Variables	Categories	No Diabetes OR (95% CI)	Diabetes OR (95% CI)	Both OR (95% CI)
Sex	Male	1	1	1
	Female	0.47 (0.38–0.60)	0.57 (0.46–0.72)	0.52 (0.45–0.62)
Age groups (Years old)	≤9	1	1	1
	50–59	NS	NS	NS
	60–69	0.67 (0.49–0.92)	0.52 (0.38–0.71)	0.59 (0.47–0.74)
	70–79	0.27 (0.19–0.40)	0.21 (0.15–0.31)	0.24 (0.19–0.31)
	≥80	0.08 (0.05–0.14)	0.07 (0.04–0.12)	0.08 (0.05–0.11)
Alcohol consumption	No	1	1	1
	Yes	1.65 (1.33–2.05)	1.63 (1.32–2.01)	1.61 (1.41–1.90)
Educational level	No studies/primary	1	1	1
	Secondary	NS	NS	1.28 (1.02–1.60)
	High education	NS	NS	NS
Living with a partner	Yes	1	1	1
	No	1.63 (1.33–1.99)	1.23 (1.01–1.51)	1.41 (1.23–1.63)
Sedentary lifestyle	No	1	1	1
	Yes	1.51 (1.22–1.86)	1.37 (1.12–1.68)	1.43 (1.24–1.66)
COPD	No	1	1	1
	Yes	1.74 (1.15–2.64)	1.72 (1.25–2.39)	1.75 (1.35–2.25)
Asthma	No	1	1	1
	Yes	0.60 (0.37–0.99)	0.60 (0.39–0.93)	0.61 (0.44–0.84)
Mental disease	No	1	1	1
	Yes	NS	1.56 (1.21–2.02)	1.29 (1.06–1.57)
High blood pressure	No	1	1	1
	Yes	0.77 (0.61–0.96)	0.78 (0.63–0.96)	0.76 (0.65–0.89)
EHISS	2014	1	1	1
	2020	NS	NS	NS
Diabetes	No	NA	NA	1
	Yes	NA	NA	1.15 (1.01–1.34)

OR: odds ratio; CI: confidence interval; COPD: Chronic obstructive pulmonary disease; EHISS: European Health Interview Surveys for Spain; NS: Not significant; NA: Not applicable.

**Table 5:** Predictors of alcohol consumption in the population participating in the European Health Interview Surveys (2014 and 2020) in Spain, according to the presence of diabetes.

Variables	Categories	No Diabetes OR (95% CI)	Diabetes OR (95% CI)	Both OR (95% CI)
Sex	Male	1	1	1
	Female	0.27 (0.23–0.31)	0.21 (0.18–0.25)	0.24 (0.21–0.27)
Age groups (Years old)	≤49	1	1	1
	50–59	NS	NS	NS
	60–69	NS	1.47 (1.09–1.97)	1.37 (1.12–1.69)
	70–79	NS	NS	NS
	≥80	0.64 (0.46–0.90)	NS	NS
Active smoking	No	1	1	1
	Yes	1.61 (1.30–2.00)	1.64 (1.33–2.02)	1.62 (1.39–1.88)
Educational level	No studies/primary	1	1	1
	Secondary	1.42 (1.15–1.75)	1.48 (1.21–1.80)	1.44 (1.25–1.66)
	High education	1.98 (1.48–2.63)	2.17 (1.60–2.94)	2.06 (1.67–2.53)
Self-rate health	Very good/good	1	1	1
	Fair/poor/very poor	0.49 (0.41–0.58)	0.63 (0.53–0.75)	0.56 (0.49–0.63)
Sedentary lifestyle	No	1	1	1
	Yes	0.64 (0.54–0.75)	0.70 (0.60–0.82)	0.67 (0.59–0.74)
Asthma	No	1	1	1
	Yes	1.45 (1.04–2.02)	NS	NS
Cardiac ischemia	No	1	1	1
	Yes	NS	0.71 (0.55–0.90)	0.82 (0.68–0.99)
Ictus	No	1	1	1
	Yes	NS	0.59 (0.42–0.83)	0.61 (0.47–0.81)
EHISS	2014	1	1	1
	2020	0.82 (0.70–0.95)	NS	NS
Diabetes	No	NA	NA	1
	Yes	NA	NA	0.70 (0.63–0.78)

OR: odds ratio; CI: confidence interval; EHISS: European Health Interview Surveys for Spain; NS: Not significant; NA: Not applicable.

## Data Availability

The anonymized EHISS datasets are freely accessible and can be downloaded by anyone on the Ministry of Health’s website. https://www.sanidad.gob.es/estadEstudios/estadisticas/EncuestaEuropea/home.htm (accessed on 24 November 2024). All other relevant data are included in the paper.

## Supplementary Materials

The following supporting information can be downloaded at: https://www.mdpi.com/article/10.3390/epidemiologia7010007/s1, Table S1: Definition of variables according to the questions included in the European Health Interview Surveys in Spain conducted in years 2014 and 2020.; Table S2: Distribution according to study variables of people with diagnosed diabetes included in the European Health Interview Surveys for Spain (EHISS) conducted in years 2014 and 2020. Table S3: Prevalence of active smoking and alcohol consumption in individuals with and without diabetes (2014–2020), including 95% Confidence Intervals (CI).

## Abbreviations

The following abbreviations are used in this manuscript:

DM	Diabetes mellitus
EHISS	European Health Interview Surveys for Spain
INE	Spanish National Statistics Institute
BMI	body mass index
COPD	chronic obstructive pulmonary disease
