# Supplementary material for: Trends of Tobacco and Alcohol Consumption Among People with Diabetes Mellitus in Spain: A Population-Based Study (2014–2020)
Source: Epidemiologia (Basel). 2026 Jan 4;7(1):7. doi: 10.3390/epidemiologia7010007 (PMC12821502; doi:10.3390/epidemiologia7010007)
Supplement: Supplementary file 1 [file epidemiologia-07-00007-s001.zip › epidemiologia-4026241-supplementary.pdf]

Table S1. Definition of variables according to the questions included in the European Health Interview Surveys in Spain conducted in years 2014 and 2020.

**Questions**	**Description and answer**	**Variables name**	**Categories**
Has your doctor told you that you are suffering from diabetes?	1. Yes<br>2. No	Diabetes	1. Case<br>2. Control
What is your sex?	1. Male<br>2. Female	Sex	1. Male<br>2. Female
How old are you?	Age in years	Age groups	1. ≤ 49<br>2. 50-59<br>3. 60-69<br>4. 60-79<br>5. ≥ 80
During the past 12 months, how often have you had alcoholic beverages of any kind (i.e. beer, wine, spirits, distilled and mixed drinks, or other alcoholic beverages)?	1. Daily or almost daily<br>2. 5-6 days per week<br>3. 3-4 days per week<br>4. 1-2 days per week<br>5. 2-3 days in a month<br>6. Once a month<br>7. Less than once a month<br>8. Not in the last 12 months, have I stopped drinking<br>9. Never or just a few sips to taste it throughout life	Alcohol consumption	1. Yes: Options 1 to 6<br>2. No: Option 7 to 9
Could you tell me if you smoke?	1. Yes, I smoke daily<br>2. Yes, I smoke, but not daily<br>3. I don't currently smoke but have smoked before<br>4. I neither smoke nor have I ever smoked regularly	Active smoking	1. Yes: Options 1 and 2<br>2. No: Options 3 and 4
What level of education have you completed?	1. Does not know how to read or write<br>2. Incomplete primary education<br>3. Complete primary education<br>4. First stage of Secondary Education, with or without a qualification<br>5. Elementary Spanish Upper Secondary Education<br>6. Upper secondary education<br>7. Intermediate vocational training or equivalent	Educational level	1. No studies/Primary: Options 1 to 3<br>2. Secondary: Options 4 to 8<br>3. High education: Options 9 and 10

	8. Advanced vocational training or equivalent<br>9. University studies or equivalent<br>10. Over university (master, PhD….)		
What is your marital status?	1. Single<br>2. Married<br>3. Widower<br>4. Separated<br>5. Divorced	Living with a partner	1. Yes: Option 2<br>2. No: options 1, 3, 4 and 5
In the past twelve month, how is your perception of your general health status?	1. Very good<br>2. Good<br>3. Fair<br>4. Bad<br>5. Very bad	Self-rated health	1. Very good/good: Options 1 and 2<br>2. Fair/poor/very poor: Options 3 to 5
1. Could you tell me how tall you are, approximately, without shoes?<br>2. Could you tell me your weight, approximately, without shoes and clothes?	Body mass index is calculated with the formulae: Weight in kg/ (Height in meters)^2	Body mass index	
Which of these possibilities best describes how often you do some physical activity in your free time?	1. I don't exercise. I occupy my free time almost completely sedentary.<br>2. I do some occasional physical or sports activity<br>3. I do physical activity several times a month<br>4. I do sports or physical training several times a week	Sedentary lifestyle	1. No: Option 1<br>2. Yes: Option 2 to 4
Has your doctor told you that you are suffering from COPD?	1.Yes<br>2.No	COPD	1. Yes<br>2. No
Has your doctor told you that you are suffering from asthma?	1.Yes<br>2.No	Asthma	1. Yes<br>2. No
Has your doctor told you that you are suffering from heart diseases (heart failure or coronary disease)?	1.Yes<br>2.No	Cardiac ischemia	1. Yes<br>2. No
Has your doctor told you that you are suffering from stroke?	1.Yes<br>2.No	Stroke	1. Yes<br>2. No
Has your doctor told you that you are suffering from cancers?	1.Yes<br>2.No	Cancer	1. Yes<br>2. No
Has your doctor told you that you are suffering from anxiety or depression?	1.Yes<br>2.No	Mental disease	1. Yes<br>2. No

Has your doctor told you that you are suffering from high blood pressure?	1.Yes<br>2.No	High blood pressure	1. Yes<br>2. No
Has your doctor told you that you are suffering from dyslipidemia?	1.Yes<br>2.No	Dyslipidemia	1.Yes<br>2.No
Has your doctor told you that you are suffering from migraine, frequent headache, chronic neck pain, or low back pain?	1.Yes<br>2.No	Pain	1.Yes<br>2.No
Has your doctor told you that you are suffering from osteoporosis?	1.Yes<br>2.No	Osteoporosis	1.Yes<br>2.No
Has your doctor told you that you are suffering from gastric or duodenal ulcer?	1.Yes<br>2.No	Gastric or duodenal ulcer	1.Yes<br>2.No
Do your gums bleed when brushing or spontaneously, or do you have missing teeth that have not been replaced with prostheses?	1.Yes<br>2.No	Gingival bleeding	1.Yes<br>2.No
Have you been involved in a traffic accident or home accident in which you were injured?	1.Yes<br>2.No	Accident	1.Yes<br>2.No

COPD: chronic obstructive pulmonary disease.

Table S2. Distribution according to study variables of people with diagnosed diabetes included in the European Health Interview Surveys for Spain (EHISS) conducted in years 2014 and 2020.

Variable	Categories	EHISS 2014 (N=1874)		EHISS 2020 (N=2053)		P value
		n	%	n	%	
**Sex**	Male	892	47.6%	1020	49.7%	0.192
	Female	982	52.4%	1033	50.3%	
**Age (Years old)**	Mean (SD)	68.1 (13.3)		69.6 (12.8)		0.064
**Age groups (Years old)**	≤49	177	9.4%	140	6.8%	< 0.001*
	50-59	274	16.6%	280	13.6%	
	60-69	493	26.3%	495	24.1%	
	70-79	512	27.3%	657	32.0%	
	≥80	418	22.3%	418	23.4%	
**Alcohol consumption**	Yes	697	37.2%	818	39.8%	0.088
**Active smoking**	Yes	284	15.2%	304	14.8%	0.761
**Educational level**	No studies/primary	604	32.2%	503	24.5%	<0.001*
	Secondary	1131	60.4%	1356	66.0%	
	High education	139	7.4%	194	9.4%	
**Living with a partner**	Yes	1013	54.1%	1112	54.2%	0.945
**Self-rated health**	Fair/poor/very poor	1240	66.2%	1231	60.0%	<0.001*
	Very good/good	634	33.8%	822	40.0%	
**Body mass index (BMI)(kg/m^2)**	Median (IQR)	28.0 (25.3 – 31.2)		27.5 (24.8 – 30.8)		0.847
**BMI category**	Underweight <18.5	8	0.5%	10	0.5%	0.009*
	Normal weight 18.5 – 24.9	385	22.3%	487	25.6%	
	Overweight 25.0 – 29.9	729	42.3%	837	43.9%	
	Obesity ≥ 30	603	35.0%	571	30.0%	
**Sedentary lifestyle**	Yes	969	51.7%	1001	48.8%	0.065
**COPD**	Yes	229	12.2%	181	8.8%	<0.001*
**Asthma**	Yes	154	8.2%	132	6.4%	0.031*
**Cardiac ischemia**	Yes	245	13.1%	232	11.3%	0.089
**Stroke**	Yes	120	6.4%	127	6.2%	0.779
**Cancer**	Yes	145	7.7%	173	8.4%	0.429
**Mental disease**	Yes	444	23.7%	388	18.9%	<0.001*
**High blood pressure**	Yes	1151	61.4%	1307	63.7%	0.147
**Dyslipidemia**	Yes	938	50.1%	1081	52.7%	0.103
**Pain**	Yes	826	44.1%	796	38.8%	<0.001 *
**Osteoporosis**	Yes	198	10.6%	182	8.9%	0.072
**Gastric/duodenal ulcer**	Yes	156	8.3%	105	5.1%	<0.001*
**Periodontal disease**	Yes	1301	69.4%	1267	61.7%	<0.001*
**Accident**	Yes	217	11.6%	170	8.3%	<0.001*

SD: standard deviation; IQR: interquartile range; COPD: chronic obstructive pulmonary disease;
*: statistically significant.

Table S3. Prevalence of active smoking and alcohol consumption in individuals with and without diabetes (2014–2020), including 95% Confidence Intervals (CI).

	Group	EHISS edition	Prevalence (%)	95% CI
**Active smoking**	Diabetes	2014	15.2	(13.5% – 16.7%)
		2020	14.8	(13.2% – 16.3%)
	No diabetes	2014	14.5	(12.9% – 16.1%)
		2020	15.8	(14.2% – 17.4%)
**Alcohol consumption**	Diabetes	2014	37.2	(35.0% – 39.3%)
		2020	39.8	(37.7% – 41.9%)
	No diabetes	2014	49.9	(47.6% – 52.2%)
		2020	47.6	(45.5% – 49.7%)

EHISS: European Health Interview Surveys for Spain; CI: confidence interval.
